# *In-ovo* echocardiography for application in cardiovascular research

**DOI:** 10.1007/s00395-023-00989-0

**Published:** 2023-05-16

**Authors:** Niklas Hegemann, Willem Bintig, Paul-Lennard Perret, Judith Rees, Alessandra Viperino, Britta Eickholt, Wolfgang M. Kuebler, Michael Höpfner, Bianca Nitzsche, Jana Grune

**Affiliations:** 1 Department of Cardiothoracic and Vascular Surgery, Deutsches Herzzentrum Der Charité (DHZC), Augustenburger Platz 1, 13353 Berlin, Germany; 2 Charité – Universitätsmedizin Berlin, Corporate Member of Freie Universität Berlin and Humboldt-Universität Zu Berlin, Institute of Physiology, Charitéplatz 1, 10117 Berlin, Germany; 3 German Center for Cardiovascular Research (DZHK), Partner Site Berlin, 10117 Berlin, Germany; 4 Charité – Universitätsmedizin Berlin, corporate member of Freie Universität Berlin and Humboldt-Universität zu Berlin, Institute of Biochemistry, Charitéplatz 1, 10117 Berlin, Germany

**Keywords:** Chicken embryo, Echocardiography, 3R, Preclinical research, Alternative methods

## Abstract

Preclinical cardiovascular research relies heavily on non-invasive *in*-*vivo* echocardiography in mice and rats to assess cardiac function and morphology, since the complex interaction of heart, circulation, and peripheral organs are challenging to mimic *ex-vivo*. While n-numbers of annually used laboratory animals worldwide approach 200 million, increasing efforts are made by basic scientists aiming to reduce animal numbers in cardiovascular research according to the 3R’s principle. The chicken egg is well-established as a physiological correlate and model for angiogenesis research but has barely been used to assess cardiac (patho-) physiology. Here, we tested whether the established *in-ovo* system of incubated chicken eggs interfaced with commercially available small animal echocardiography would be a suitable alternative test system in experimental cardiology. To this end, we defined a workflow to assess cardiac function in 8–13-day-old chicken embryos using a commercially available high resolution ultrasound system for small animals (Vevo 3100, Fujifilm Visualsonics Inc.) equipped with a high frequency probe (MX700; centre transmit: 50 MHz). We provide detailed standard operating procedures for sample preparation, image acquisition, data analysis, reference values for left and right ventricular function and dimensions, and inter-observer variabilities. Finally, we challenged incubated chicken eggs with two interventions well-known to affect cardiac physiology—metoprolol treatment and hypoxic exposure—to demonstrate the sensitivity of *in-ovo* echocardiography. In conclusion, *in-ovo* echocardiography is a feasible alternative tool for *basic* cardiovascular research, which can easily be implemented into the small animal research environment using existing infrastructure to replace mice and rat experiments, and thus, reduce use of laboratory animals according to the 3R principle.

**Supplementary Information:**

The online version contains supplementary material available at 10.1007/s00395-023-00989-0.

## Introduction

Today, basic research on the cardiac ventricles, their pathologies (e.g., heart failure) and potential secondary end organ damage (e.g., to the pulmonary circulation) are almost exclusively conducted using small animals, such as mice and rats. To this end, echocardiography for reliable assessment of cardiac structure and function has undergone tremendous technical advances over the last decades and has become a mainstay of preclinical cardiovascular characterization in small animals using ultra-high-frequency transducers with excellent spatio-temporal resolution [3, 14]. Echocardiography also benefits from generally good availability in cardiovascular research labs or centres and user-friendliness and has, therefore, become the gold standard for monitoring cardiac function in rodents *in-vivo*.

We have previously shown that incubated chicken eggs (iCE) offer a promising alternative to small animal models for investigation of tumor biology, novel anti-cancer treatments, and angiogenesis [27, 28, 34]. The heart is the first functional organ of the chicken embryo and starts beating as early as 33 h post-incubation [5, 38]. Extensive growth leads to a reshaping of the early heart tube into a complete four-chambered heart in only 8 days [23]. Due to their fast development, good accessibility of the cardiovascular system, and the possibility of high-throughput analysis, we propose to implement iCEs as experimental tool in basic research with a focus on the cardiovascular system, providing rapid results in a short time span of roughly 2 weeks [32, 37]. Importantly, as nociception in birds is similar to that in mammals and the surface exposed to the transducer is not innervated, the experiments are not associated with pain perception by the embryo [9, 21]. Furthermore, avian embryos can serve as versatile alternative model as they are not classified as animal experiments prior to hatching according to the European legislation for animal welfare (Directive 2010/63/EU).

Here, we comprehensively evaluated the feasibility of *in-ovo* echocardiography in iCEs as an alternative model to *in-vivo* small animal echocardiography and provide an easily replicable and detailed standard operating procedure (Supplementary Material 1). To this end, we performed morphological and functional echocardiographic assessments of the chick embryo’s heart between embryonic day (ED) 8 and ED 13 and assessed cardiac effects of sympathetic tone inhibition or sub-chronic hypoxia exposure to evaluate the sensitivity of our model and to detect alterations in cardiac structure and function. In addition, we conducted inter-observer analyses to evaluate reproducibility of data analysis between an expert, advanced and novice echocardiographer. Our results indicate methodological feasibility, robustness, and sensitivity of *in-ovo* echocardiography. Assessed reference values can prospectively serve as guide for future users from the basic research community in the cardiovascular field.

## Methods

### *In-ovo* chick embryo model

Fertilized, specifically pathogen-free eggs of white leghorn chicken (*Gallus gallus*) were obtained from VALO BioMedia GmbH (Cuxhaven, Germany) and stored at 14 °C for maximum 10 days until start of the incubation. Embryonic development was initiated by placing the eggs in a horizontal position into a humidified (> 58%) egg incubator at 37.8 °C (Type Janoel JN8-48, China). At day 3 of egg development, small holes were pierced into the egg poles and 6 ml of albumin were removed using a 20 ml syringe. This process was controlled using a gooseneck lamp to illuminate the iCE through the eggshell. The chicken embryos were then again incubated and continued developing until ED 8 to ED 13 when final experiments were performed.

### Echocardiography

**Fig. 1:**
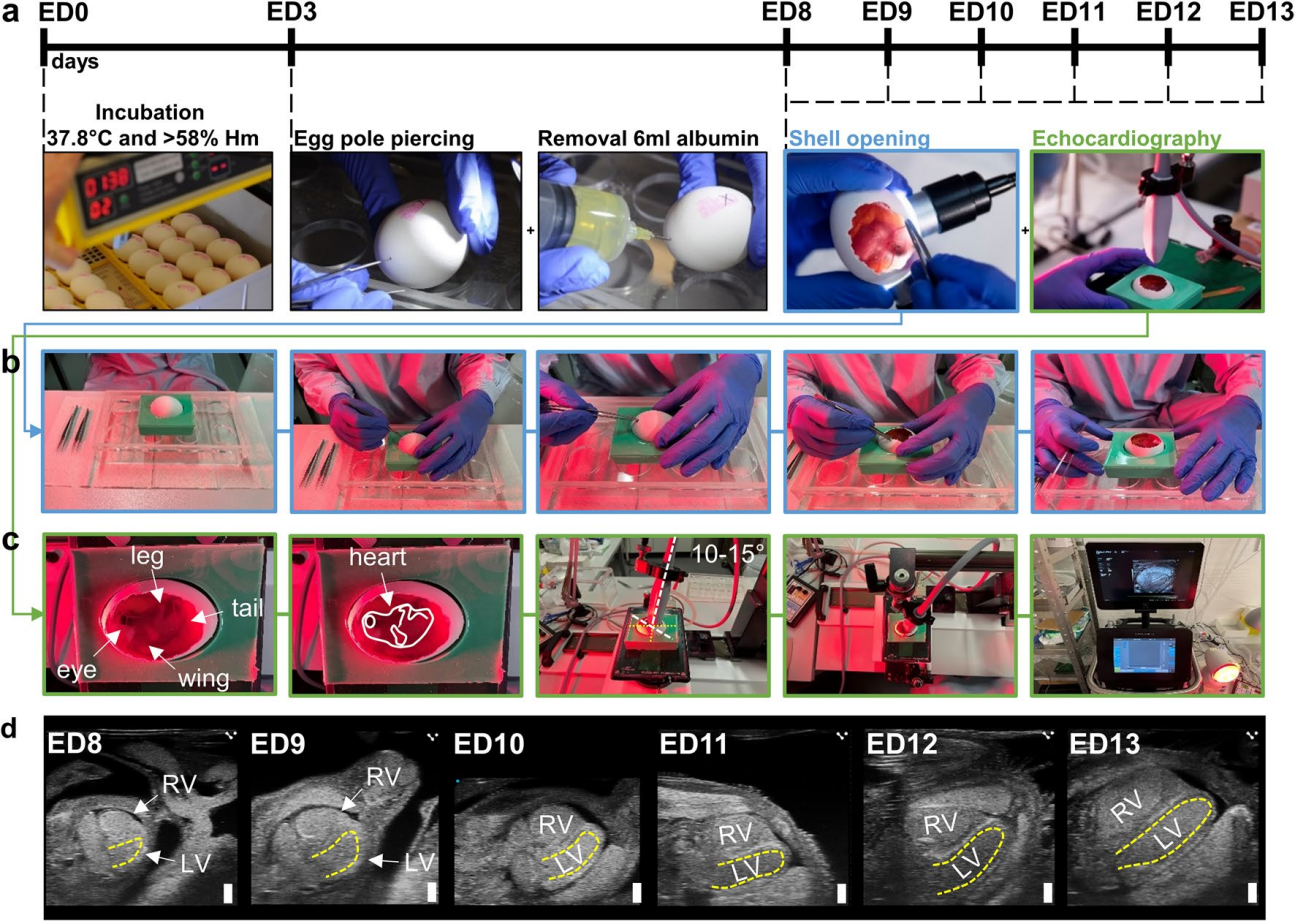
iCE incubation scheme and experimental outline. **a** iCEs were incubated starting ED 0 in a special incubator at 37.8 °C and > 58% humidity (Hm). On ED 3, the egg poles were pierced, and 6 ml of albumin were removed to lower the chorioallantoic membrane. **b, c** On ED 8 to ED 13 iCEs were subject to transthoracic echocardiography *in-ovo*. **d** Representative echocardiographic B-Mode images in the modified five-chamber view (^mod^5CV) of iCEs on ED 8 to ED 13. Yellow contours depict left-ventricular endocardial borders. *RV*  right ventricle, *LV*  left ventricle. Scale bar = 1 mm

**Fig. 2:**
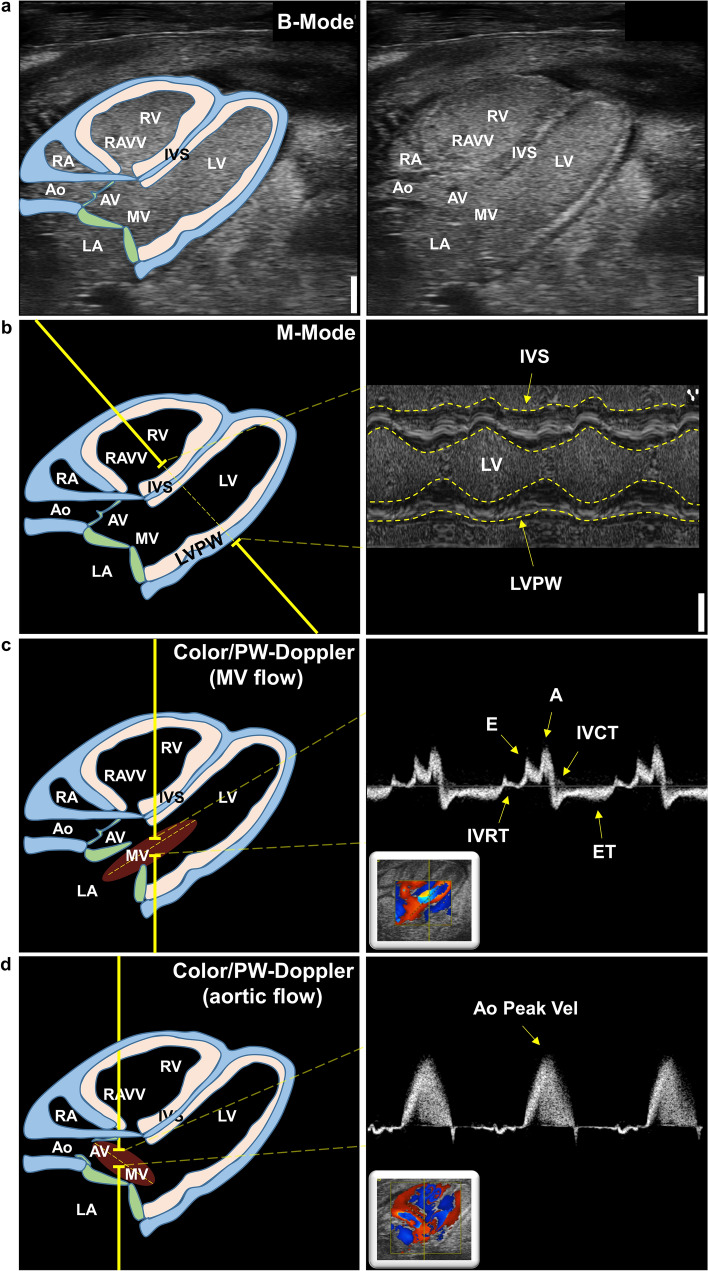
Echocardiographic image acquisition in iCEs. **a** Representative B-Mode image in the modified five-chamber view (^mod^5CV) with schematic outline of relevant structures (left panel). **b** Schematic B-Mode image of the ^mod^5CV with instructions for left-ventricular (LV) M-Mode imaging (left panel) and representative M-Mode tracing of interventricular septum, LV lumen, and LV posterior wall (right panel). **c** Schematic B-Mode image of the ^mod^5CV with instructions for color/pulsed-wave Doppler of the trans-mitral inflow (left panel) with representative color Doppler and flow profile (right panel). **d** Schematic B-Mode image of the ^mod^5CV with instructions for color/pulsed-wave Doppler of the aortic flow (left panel) with representative color Doppler and flow profile (right panel). *Ao*  aorta, *AV*  aortic valve, *MV*  mitral valve, *LA*  left atrium, *LV*  left ventricle, *RA*  right atrium, *RV*  right ventricle, *RAVV*  right atrioventricular valve, *IVS*  interventricular septum, *LVPW*  left-ventricular posterior wall, *E*  early (passive) diastolic inflow, *A*  active inflow due to atrial contraction, *IVRT*  isovolumic relaxation time, *IVCT*  isovolumic contraction time, *ET*  ejection time, *Ao Peak Vel*  peak aortic velocity. Scale bar = 1 mm

Evaluation of cardiac function and morphology by echocardiography was performed on ED 8 to ED 13 (Fig. 1a). The eggshell was cautiously opened with fine forceps to the maximal possible extent without injuring the chorioallantoic membrane (CAM) (Fig. 1b). Two-hundred fifty µl of pre-heated (~ 37 °C) calcium/magnesium-free PBS were added on top of the CAM to protect it from drying and to act as coupling medium for the subsequent echocardiography. The eggs were placed into a self-made silicone mold on a pre-warmed heating table at 38–40 °C. To additionally assure physiological temperatures, a heating lamp was used and temperatures on the CAM were monitored using an infrared thermometer, aiming for a 34–36 °C window. For state-of-the-art imaging a commercially available Vevo 3100 small animal ultrasonography system was used in conjunction with a high-frequency MX700 (29–71 MHz; centre transmit 50 MHz) linear array transducer (all Fujifilm Visualsonics Inc., Canada) and the “Mouse Vascular” application preset. For highest reproducibility and accuracy, we used a railing system to mount the transducer and guide it within the egg only by the micromanipulators. Prior to imaging we visually assessed the location of the chick embryo within the egg to angle the transducer according to the suspected position of the heart to achieve a modified 5 chamber view (^mod^5CV), defined by a 45° angle of the apex (Fig. 2a). To ensure validity and integrity of our imaging approach, we employed the following inclusion criteria for image acquisition: (i) heart rate at the time of recording at least 160 beats per minute (except for metoprolol experiments), (ii) clear delineation of endocardial borders, (iii) absence of imaging artifacts masking relevant image areas, and (iv) no apical foreshortening (Supplementary Fig. 1). Three B-Mode cine loops were recorded to assess cardiac volumetry and function (Fig. 2a). Next, the anatomical M-Mode was placed centrally across the greatest left ventricular (LV) diameter and again three cine loops were recorded for analysis of cardiac dimensions, LV mass (1.053*[LV diastolic inner diameter + LV diastolic posterior wall thickness + diastolic interventricular septal thickness)^3^–LV diastolic inner diameter^3^]), and function (Fig. 2b). The color Doppler was used to detect blood flow across the mitral valve (MV)—usually just above the tip of the opened valve leaflets—and in the LV outflow tract/aorta (Fig. 2c, d). Color Doppler imaging was used to identify the correct acquisition plane and flow profiles were recorded using the pulsed-wave Doppler. To assess right ventricular (RV) function and dimensions, M-Mode recordings were performed in the previously described ^mod^5CV (Fig. 6a). Afterwards, the image focus was shifted to the pulmonary artery (PA) using the micromanipulators (Fig. 6b). PA flow, identified by Color Doppler as flow exiting the RV, was subsequently measured by pulsed-wave Doppler to assess pulmonary acceleration time, ejection time, peak flow and velocity time integral (Fig. 6c). All recordings were stored raw in the DICOM format for offline data analysis using the dedicated Vevo LAB software V5.6.1 (Fujifilm Visualsonics Inc., Canada). Data analysis was performed according to common standards. iCEs with low CAM temperature (< 34 °C) and consequently HR (< 160 beats per minute) were excluded from analysis for reference values. For all Doppler recordings, a target flow angle of approximately 45–50° was set. Recordings with flow angles above 60° were excluded from analysis.

### Metoprolol treatment

After echocardiographic baseline readings, a subset of iCEs was subject to treatment with the β1-adrenoceptor selective blocker metoprolol (metoprolol tartrate; Supelco Inc.). A single dose of 400 µg dissolved in 200 µl 0.9% sodium chloride (2.9 mM) was pipetted onto the CAM. Controls were treated with the same volume of 0.9% saline. Eggs were placed back into the incubator for 10 min before performing post-treatment echocardiographic recordings.

### Hypoxic incubation

ED 8 iCEs were placed into a conventional cell culture incubator with 15% O_2_ and 1% CO_2_ until ED 13 (Fig. 9a). In parallel, a batch of iCEs of the same charge was incubated under normoxia (room air).

### Inter-observer analysis

To assess agreement rates of echocardiographic analyses in the iCEs between users with different levels of expertise, we performed conventional Blant–Altmann analyses for a single parameter each from B-Mode, M-Mode, and pulsed-wave Doppler recordings (Fig. 10). Each observer performed these measurements in the same images from a total of 10 iCEs (5 iCEs on ED 9 and 5 iCEs on ED 13 of incubation). The expert user had more than 4 years of experience in small animal echocardiography, the advanced user more than 2 years, and the novice less than 1 year.

### LV geometry assessment in iCE and adult mice

For comparison of LV geometry in iCEs and adult mice, we performed analysis of eccentricity and ellipticity (Supplementary Fig. 2). On this account, we evaluated LV dimensions of five iCE on ED 13 and in five 22-week-old adult C57BL/6 mice. Mice experiments were approved by the local authorities (LAGeSo) under the registration G0017/20. Parasternal long-axis B-Mode images from mice were evaluated and acquired as previously described [12, 13].

### Euthanasia and gravimetric analysis

After *in-ovo* echocardiography, iCEs were removed from the eggs and immediately euthanized by decapitation using surgical scissors. The body weight including the head was recorded. Subsequently, the thorax was opened and the heart was carefully excised for weight documentation.

### Statistics

All data are presented as mean ± SEM. Data were investigated for Gaussian distribution by Shapiro–Wilk normality test. Accordingly, data were further tested by unpaired (or paired for metoprolol treatment data) student’s *t* test or Mann–Whitney test. Statistical significance was assumed for *P* > *0.05*. Correlation analysis was performed by Pearson’s correlation and simple linear regression. Inter-observer analysis was performed using conventional Blant–Altmann plots with indication of 95% limits of agreement (LoA) and bias.

## Results

### iCE preparation and echocardiographic image acquisition

Experiments were performed in non-anesthetized, temperature-controlled iCEs. Removal of albumin on ED 3 by piercing of small holes caused the developing CAM to detach from the eggshell and lower itself into the allantoic cavity (Fig. 1a). Incubation of iCEs was continued until ED 8 to ED 13 when echocardiographic assessment was performed. On the respective day of echocardiography, eggshells were opened to the maximal possible extent without injuring the CAM (Fig. 1b). iCEs were placed horizontally into a self-made silicone mold illuminated by a heating lamp. Approx. 70% of iCEs are in a lateral-to-supine position and the head is positioned towards the blunt end of the horizontally placed egg (Fig. 1c). Approx. 20% of iCEs are in a lateral-to-supine position, but the head is positioned towards the pointy end of the horizontally placed egg, triggering a re-orientation of the egg by 180° for image acquisition. 10% of iCEs are positioned in an abdominal position, preventing adequate accessibility of the heart for echocardiography. For state-of-the-art imaging, a commercially available small animal ultrasonography system was used in conjunction with a high-frequency linear array transducer. Using a railing system, the transducer was carefully lowered into the egg, angling the probe at an angle of approx. 10°–15° pointing towards the apex of the heart. The transducer position was adjusted using the micromanipulators under visual control of the ultrasound image until a modified five-chamber view (^mod^5CV) was achieved covering the LV, left atrium (LA), RV, right atrium (RA) and aorta (Ao) (Figs. 1d, 2a). B-Mode cine loops covering the entire heart revealed good visibility of anatomical landmarks of a fully developed heart (Fig. 2a, Suppl. Vid. 1, 2). Next, the anatomical M-Mode was placed centrally across the largest diameter of the LV to visualize LV wall motion and dimensions (Fig. 2b). Color Doppler-guided aortic valve and MV flow profiles were assessed at the tip of the opened valve leaflets using pulsed-wave-Doppler echocardiography (Fig. 2c, d). In summary, these results demonstrate accessibility, feasibility, and easy-to-do application of standard ultrasonic imaging modalities to iCEs.

### Cardiac dimensions of iCEs continuously grow after ventricular septation

**Fig. 3:**
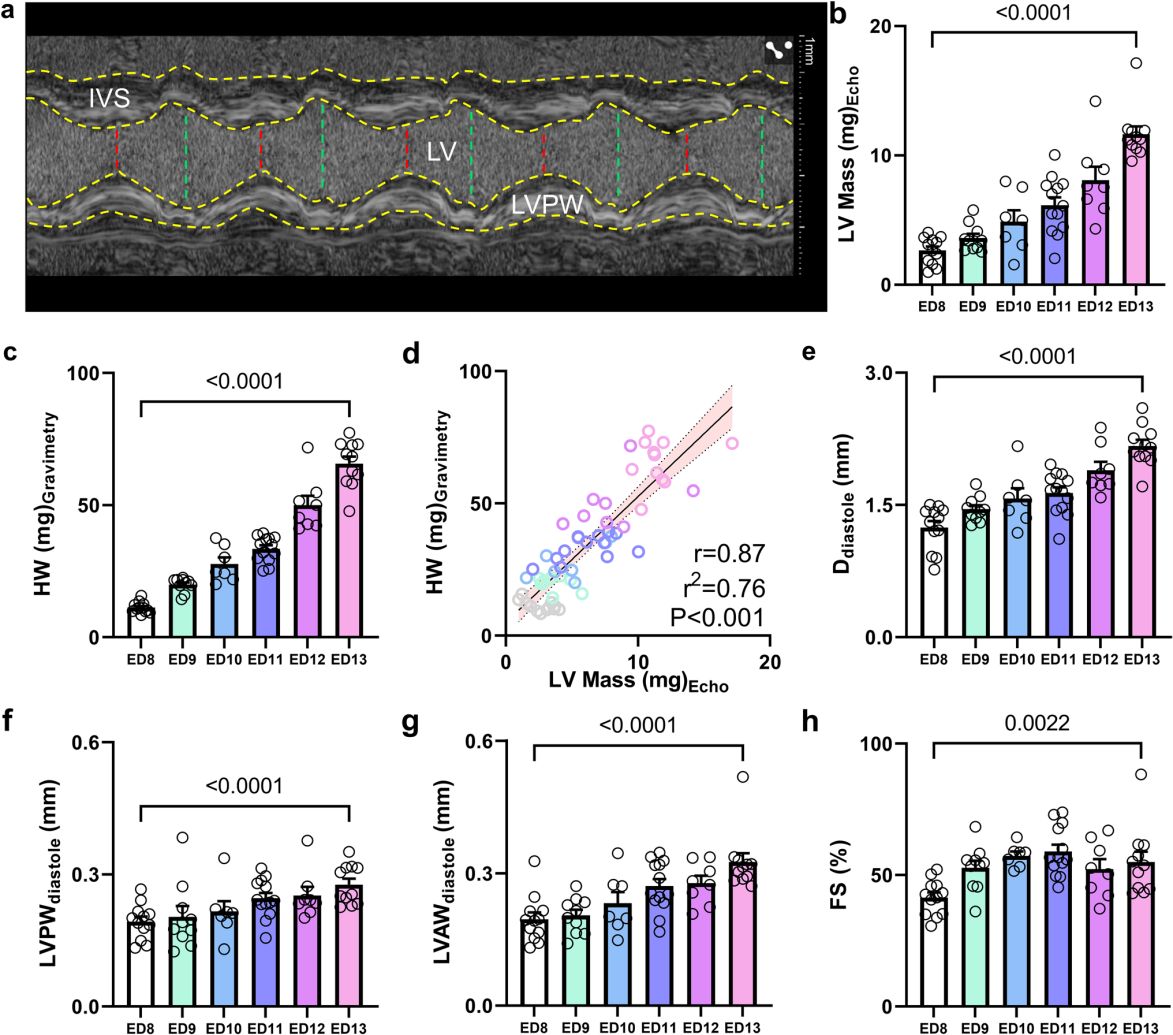
M-Mode imaging of the left ventricle on ED 8 to ED 13. **a** Representative M-mode tracing of the left ventricle (LV) with interventricular septum (IVS), LV lumen and LV posterior wall (LVPW). Yellow contours indicate myocardial borders. LV diameters in diastole are shown in green, systole in red. **b** Echocardiography-derived calculated LV mass. **c** Gravimetrically measured heart weight (HW). **d** Pearson’s correlation between the two measurements from (**b)** and (**c)**. **e** LV diameter (D) in diastole. **f** LVPW thickness in diastole. **g** LV anterior wall (LVAW) thickness in diastole. **h** Fractional shortening of the LV (%). *N* = 7–13 per group. Data are presented as mean ± SEM. Statistical analysis was performed for ED 8 vs ED 13 using student’s *t* test or, where applicable, Mann–Whitney test

Echocardiographic M-Mode imaging of the LV revealed good image quality (Fig. 3a) with proper delineation of myocardial border regions. Formula-based estimation of LV mass revealed a 4.4-fold increase from ED 8 to ED 13 (Fig. 3b). In line, gravimetrically measured total heart weights similarly showed a steady gain from ED 8 to ED 13 (Fig. 3c). Both measures, LV mass and heart weights, showed strong correlation (r = 0.87, *P* < *0.001*), indicating good agreement between gravimetry and echocardiography derived values (Fig. 3d). LV inner diameter during diastole rose from ED 8 by approx. 60% on ED 13 (Fig. 3e). LV anterior and posterior wall thicknesses and LV fractional shortening (FS) all indicated an increase from ED 8 to 13 (Fig. 3f–h), implying that after complete formation of cardiac anatomy on ED 8, LV dimensions continue to grow as part of further embryonic development.

### LV systolic function plateaus and myocardial compliance increases in iCEs after ED 8

**Fig. 4:**
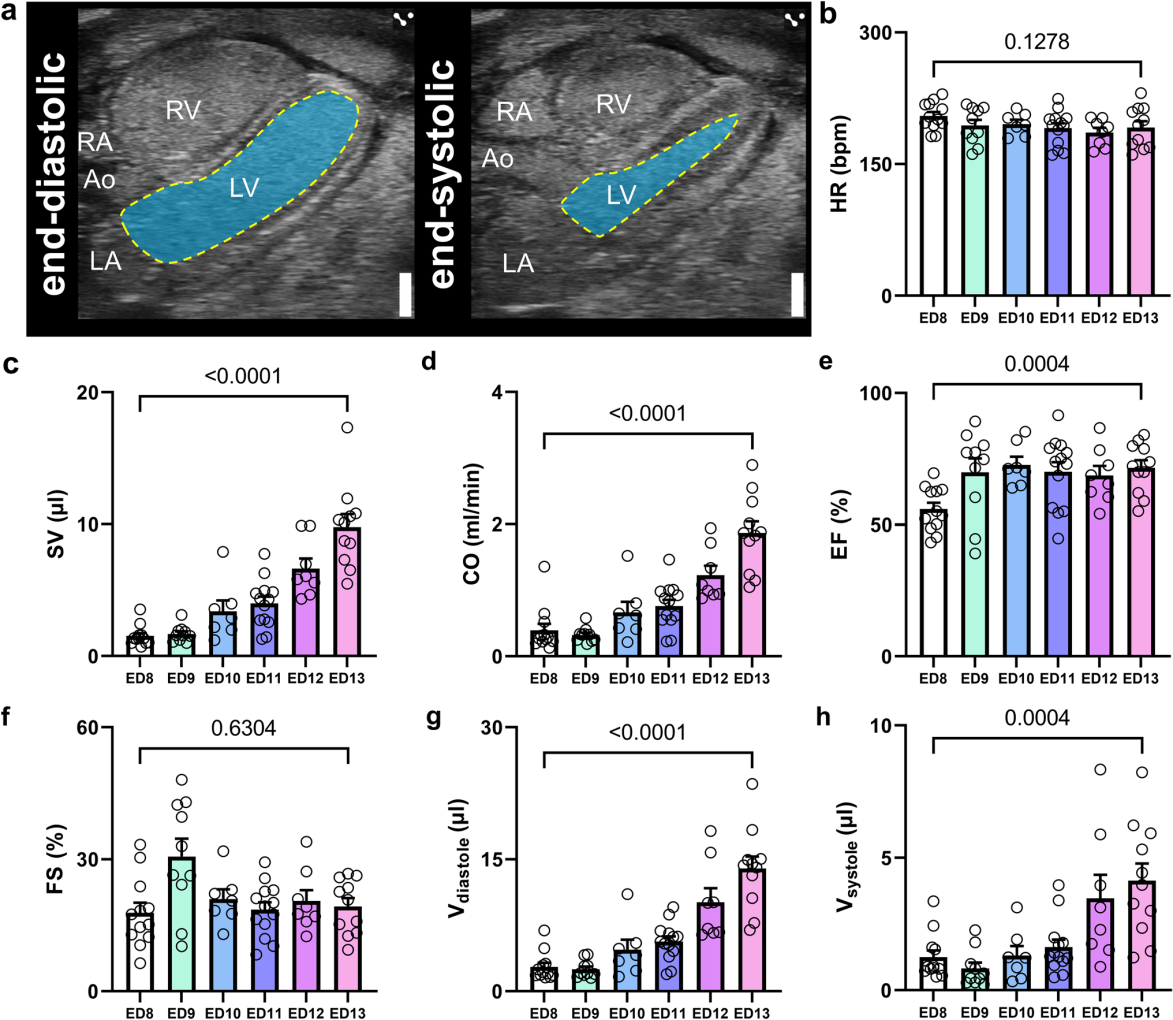
B-Mode imaging of the left ventricle in the modified five-chamber view (^mod^5CV) on ED 8 to ED 13. **a** Representative B-Mode modified five chamber views (^mod^5CV) with tracing of the LV endocardial borders (yellow) and LV area (blue) in systole and diastole. **b** Heart rate (HR) in beats per minute (bpm). **c** Stroke volume (SV). **d** Cardiac output (CO). **e)** Left-ventricular ejection fraction (LVEF). **f)** LV fractional shortening (FS). **g** LV end-diastolic volume (*V*_diastole_). **h** LV end-systolic volume (*V*_systole_). *Ao*  aorta, *LA*  left atrium, *LV*  left ventricle, *RA*  right atrium, *RV*  right ventricle. *N* = 7–13 per group. Data are presented as mean ± SEM. Statistical analysis was performed for ED 8 vs ED 13 using student’s *t* test or, where applicable, Mann–Whitney test

**Fig. 5:**
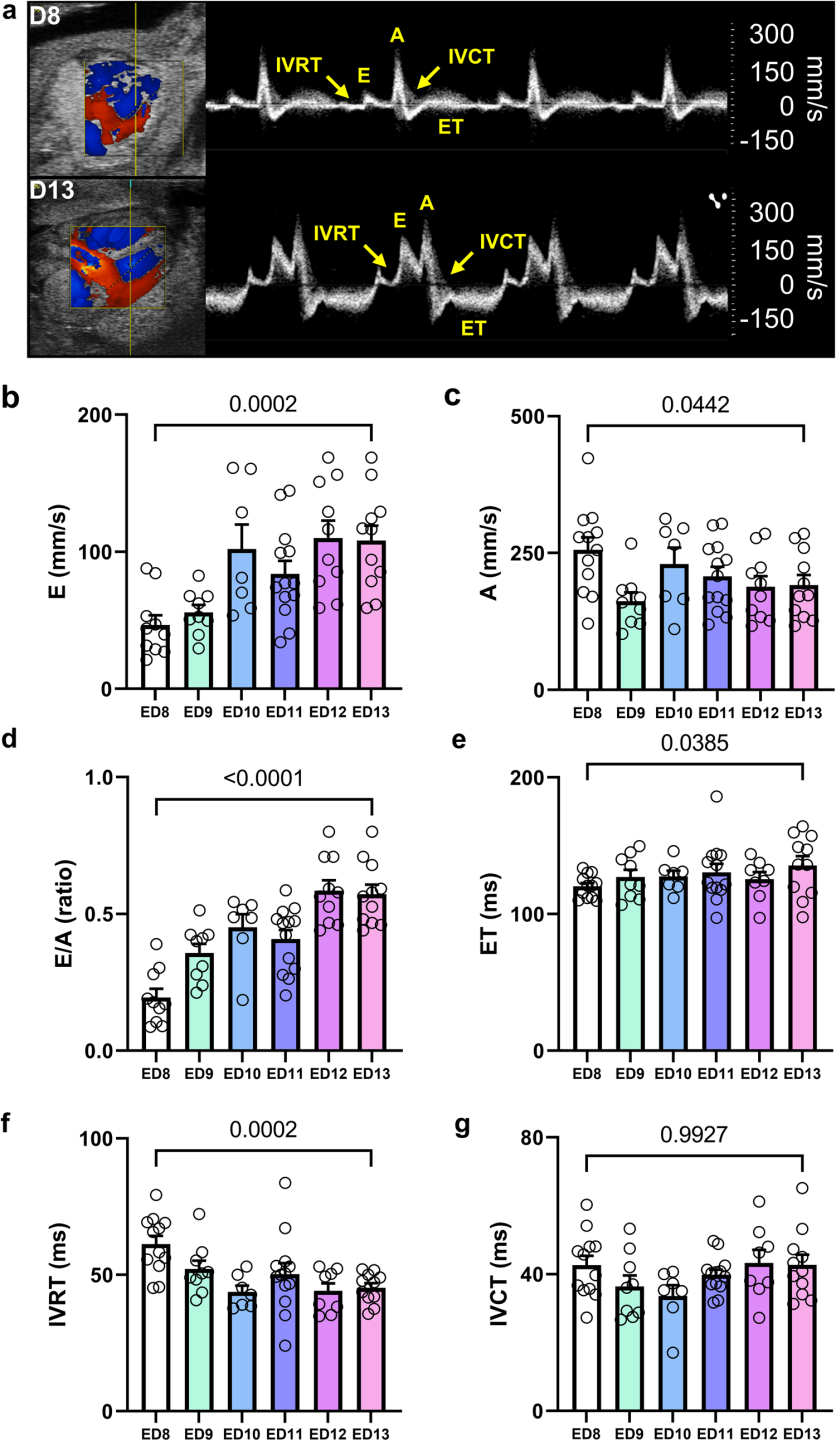
Pulsed-wave Doppler analysis of transmitral blood flow. **a** Representative transmitral flow profiles at ED 8 and ED 13 of incubation. **b** Early diastolic inflow – E wave (**E**). **c** Late diastolic inflow due to atrial contraction—A wave (**A**). **d** E/A ratio. **e** Ejection time (ET) during which blood is ejected towards the aorta. **f** Isovolumic relaxation time (IVRT). **g** Isovolumic contraction time (IVCT). *N* = 7–13 per group. Data are presented as mean ± SEM. Statistical analysis was performed for ED 8 vs ED 13 using student’s *t* test or, where applicable, Mann–Whitney test

Functional analysis of the LV was carried out using B-mode cine loops of ^mod^5CVs (Fig. 4a). LV volumetric and functional data were retrieved from LV tracings in end-diastole and end-systole defined as largest and smallest LV dimensions over the cardiac cycle. Heart rate (HR) remained stable during the observation period from ED 8 on, in line with previous reports (Fig. 4b) [6]. Stroke volume (SV) rose approximately 6.5-fold from ED 8 to ED 13 and in line, cardiac output (CO) more than quadrupled in the same time frame (Fig. 4c, d). LV ejection fraction (EF) and FS were constant from ED 9 onwards throughout ED 13 (Fig. 4e, f). Calculation of LV end-diastolic and end-systolic volumes using a modified Simpson’s monoplane method of disks [20] revealed both to continuously increase between ED 8 to ED 13 (Fig. 4g, h). Assessment of trans-mitral flow patterns revealed marked increases in the flow velocity of the early diastolic inflow (E wave), whereas the late diastolic flow (A peak) declined from ED 8 to ED 13 (Fig. 5a–c). As a result, the E/A ratio, a well-established echocardiographic marker of diastolic function, significantly increased over the observation period (Fig. 5d). In line, LV ejection time (ET), a systolic time interval indicating LV contractility, increased significantly from ED 8 to 13, while the LV isovolumic relaxation time (IVRT), a diastolic time interval indicating relaxation, shortened over the same time period (Fig. 5e, f). The isovolumic contraction time (IVCT) did not differ throughout our observations (Fig. 5g). Taken together, LV systolic function stabilizes after ED 9 of embryonic development. Diastolic relaxation and compliance increased in the assessed time frame, likely due to developmental changes of the myocardial compliance as known for human embryonic development [17, 33, 39].

### RV dimensions and function increase, while PA hemodynamics remain stable between ED 8 and ED 13

**Fig. 6:**
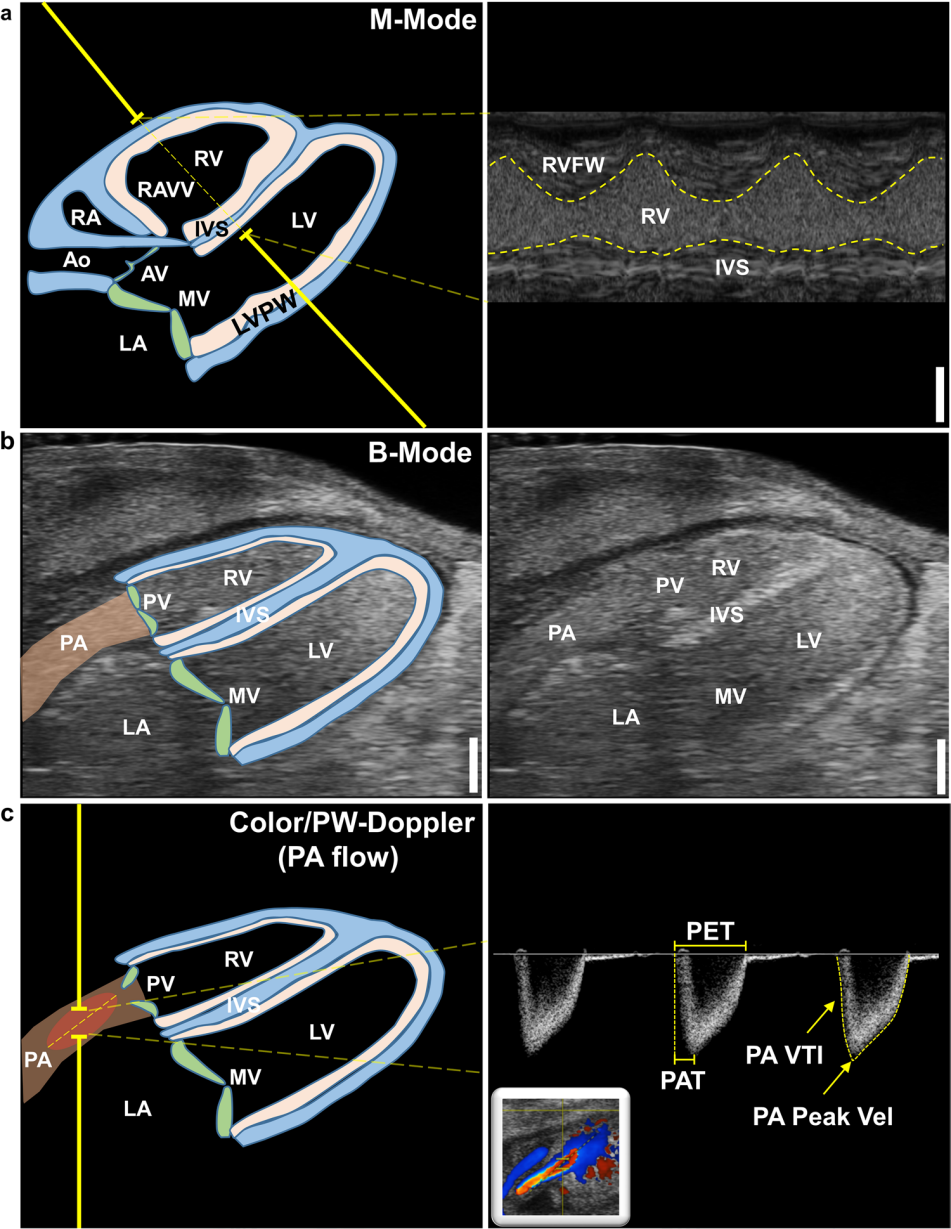
Echocardiographic right-ventricular image acquisition in iCEs. **a** Schematic ^mod^5CV as described earlier in Fig. 2 with left ventricle (LV), left atrium (LA), right ventricle (RV), right atrium (RA) and aorta (Ao) (left panel) and RV M-Mode tracing (right panel). **b** B-Mode image of the ^mod^5CV with focus shifted to the pulmonary artery (PA). **c** Schematic B-Mode image of the ^mod^5CV with focus shifted to the PA with instructions for color/pulsed-wave Doppler of the pulmonary flow (left panel) with representative color Doppler and flow profile (right panel). *Ao*  aorta, *AV*  aortic valve, *MV*  mitral valve, *LA*  left atrium, *LV*  left ventricle, *RA*  right atrium, *RV*  right ventricle, *RAVV*  right atrioventricular valve, *IVS*  interventricular septum, *LVPW*  left-ventricular posterior, *PV*  pulmonary valve, *PA*  pulmonary artery, *RVFW*  RV free wall, *IVS*  interventricular septum, *LV*  left ventricle, *MV*  mitral valve, *LA*  left atrium, *PET*  pulmonary ejection time, *PAT*  pulmonary acceleration time, *PA Peak Vel* = pulmonary peak velocity, *PA VTI*  PA velocity time integral. Scale bar = 1 mm

**Fig. 7:**
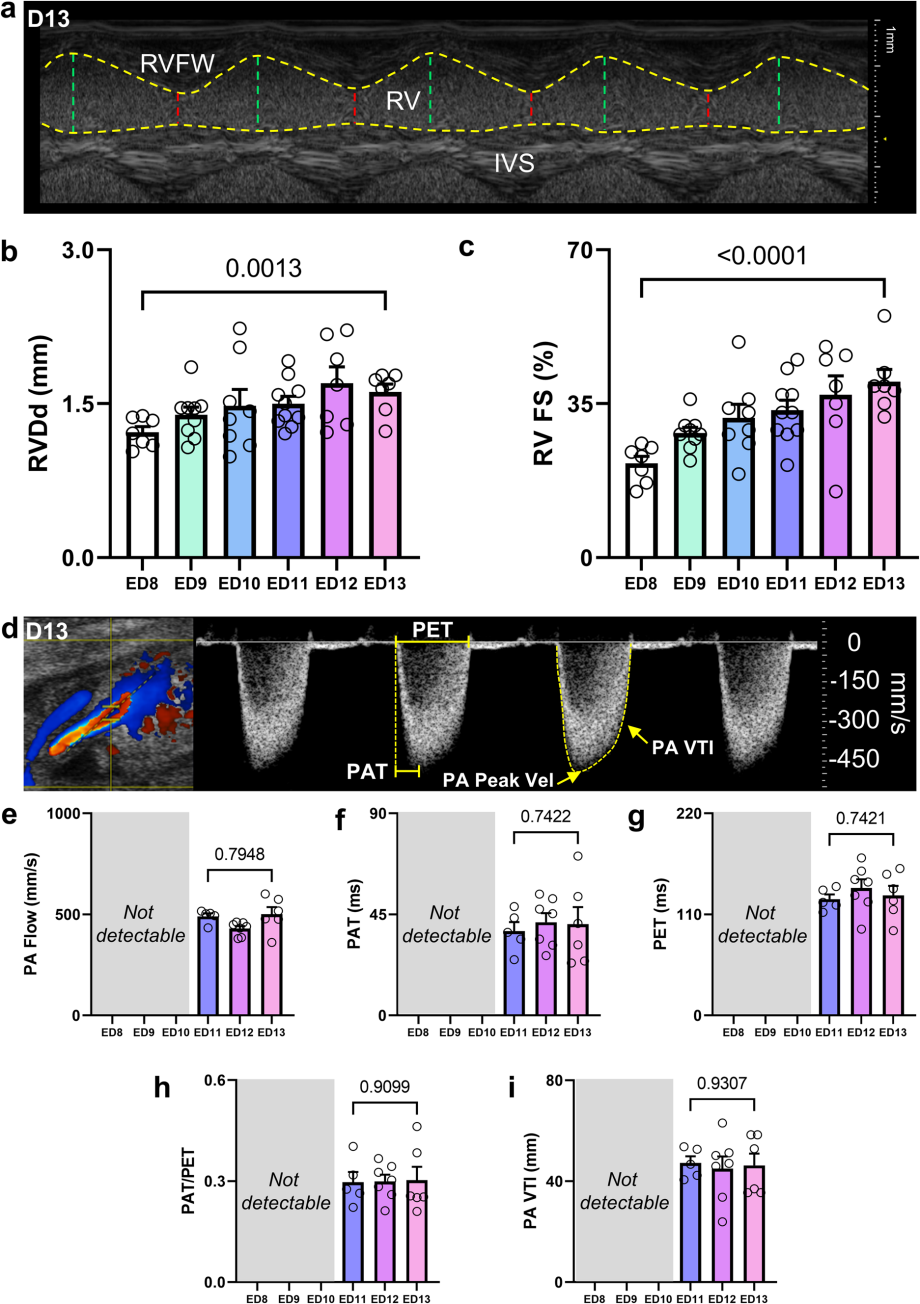
Echocardiographic right-ventricular and pulmonary artery measurements in iCEs ED 8 to ED 13. **a** Representative right-ventricular (RV) M-Mode. **b** RV diastolic diameter (RVDd). **c** RV fractional shortening (RV FS). **d** Representative pulmonary artery (PA) flow with pulmonary ejection time (PET), pulmonary acceleration time (PAT) and peak pulmonary flow velocity (PA peak Vel). **e** PA peak flow velocity, **f** PAT. **g** PET **h** PAT/PET ratio and **i)** PA Velocity Time Integral (VTI). *N* = 7–10 for (**b**) and (**c**); N = 5–7 fpor (**e**) to (**h**). Data are presented as mean ± SEM. Statistical analysis in (**b**) and (**c**) was performed for ED8 vs ED 13 using student’s *t* test or, where applicable, Mann–Whitney test. *RV*  right ventricle, *PA*  pulmonary artery, *RVFW*  RV free wall, *PET*  pulmonary ejection time, *PAT*  pulmonary acceleration time, *PA Peak Vel*  pulmonary peak velocity, *PA VTI*  PA velocity time integral

Functional and dimensional analyses of the RV were performed starting out from the ^mod^5CV, collecting RV M-Mode images (Fig. 6a). Then, using the micromanipulators and rotating the ultrasound transducer counterclockwise, the image focus was adapted to visualize the PA, then collecting pulsed wave-Doppler recordings of the PA flow directly behind the pulmonic valve within the PA using Color-Doppler guidance (Fig. 6b, c). RV chamber dimensions and FS both increased by approx. 30% between ED 8 to ED 13 (Fig. 7a–c). PA flow could only be properly assessed onwards from ED 11 as proper visualization was often unsuccessful on earlier EDs, likely due to early stage development. Between ED 8 and ED 13, none of the measured parameters such as PA flow velocity, PA acceleration time (PAT), PA ejection time (PET), PAT/PET ratio or PA velocity time integral (VTI) changed (Fig. 7d–i).

### Acute metoprolol treatment exerts negative chronotropic and inotropic effects in iCEs

**Fig. 8:**
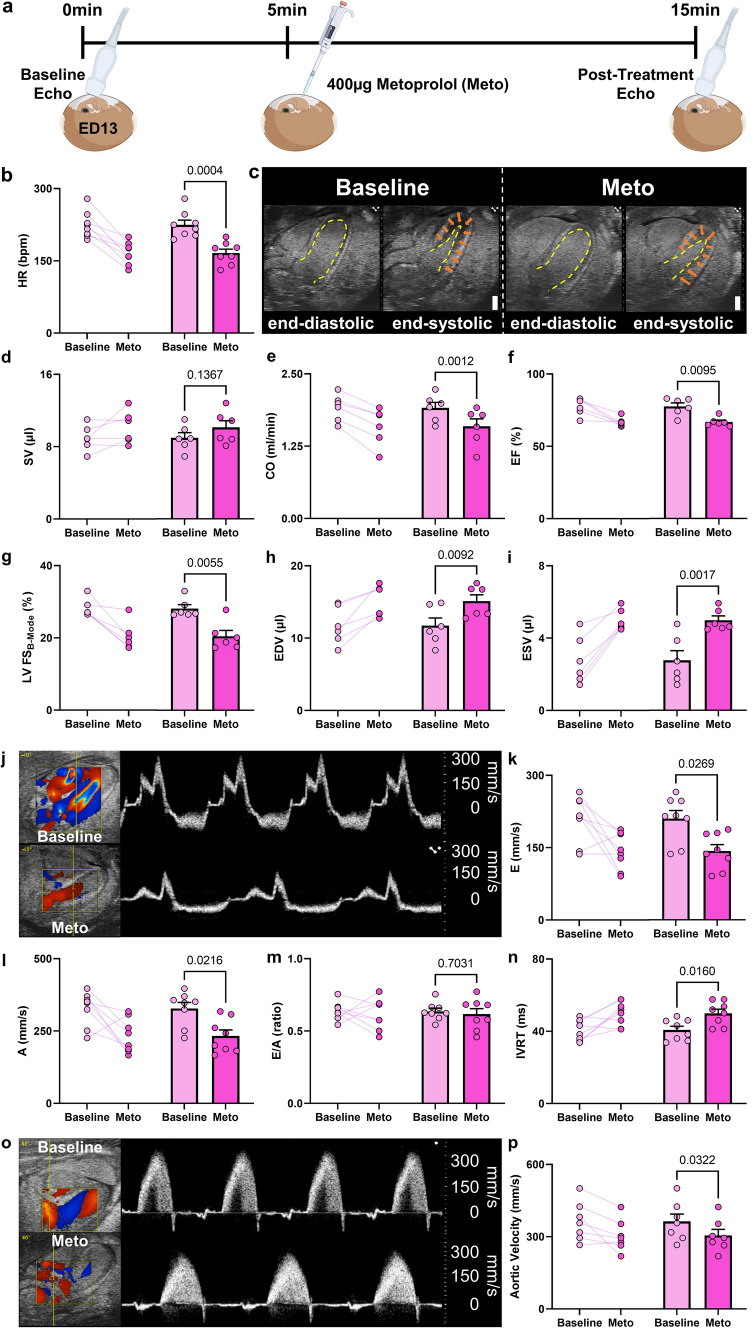
Echocardiographic evaluation of acute β_1_-selective adrenergic inhibition by metoprolol in the iCE on ED 13. **a** Experimental outline. Eggs were incubated for 13 days as described earlier. After acquisition of baseline images (approx. 5 min duration), animals were treated with a single 400 µg dose of metoprolol pipetted onto the chorioallantoic membrane. After 10 min, post-treatment image acquisition was performed. **b** Representative B-Mode five-chamber view images at baseline and after metoprolol (Meto) treatment showing left ventricular endocardial borders (yellow dotted line) at end-diastole and end-systole. Orange arrows indicate myocardial contraction. Scale bar = 1 mm. **c** Stroke volume (SV). **d** Heart rate in beats per minute (bpm). **e** Cardiac output (CO). **f** Left-ventricular ejection fraction (EF). **g** LV fractional shortening (acquired in B-Mode) (FS_B-Mode_). **h** End-diastolic volume (EDV). **i** End-systolic volume (ESV). **j** Representative color (left) and pulsed-wave Doppler recordings (right) of the transmitral blood flow from eggs at baseline and after metoprolol treatment. **k** Early diastolic inflow – E wave (E). **l** Late diastolic inflow due to atrial contraction – A wave (A). **m** E/A ratio. **n** Isovolumic relaxation time (IVRT). **o** Representative color (left) and pulsed-wave Doppler recordings (right) of the aortic blood flow at baseline and after metoprolol treatment. **p** Peak aortic velocity. *N* = 6–8 per group. Left panels represent paired data at baseline and after Meto treament, right panels present data as mean ± SEM. Statistical analysis was performed by paired student’s *t* test

To assess whether *in-ovo* echocardiography is sensitive enough to detect acute pharmacological effects on cardiac function, we treated iCEs with metoprolol, a β-1 adrenoceptor selective antagonist (400 µg single dose) and assessed cardiac function at baseline and 10 min after metoprolol treatment (Fig. 8a). Metoprolol is among the most commonly prescribed cardiovascular drugs for the treatment of coronary artery disease and hypertension and reduces HR and CO [26]. Our results revealed the expected significant decline in HR after metoprolol treatment (Fig. 8b). B-Mode cine-loops of the ^mod^5CV showed increased end-diastolic and end-systolic LV and RV chamber dimensions in iCEs after metoprolol treatment in line with increased ventricular filling at lower HR (Fig. 8c, Suppl. Vid. 3, 4). CO decrease by metoprolol was mainly caused by lowering HR, while SV remained largely unchanged (Fig. 8d, e). In line with unchanged SVs against increased chamber dimensions, EF and FS declined with metoprolol treatment by 14% and 29%, respectively (Fig. 8f, g). While LV volumes significantly increased, pulse-wave-Doppler flow profiles decreased after metoprolol treatment (Fig. 8h–j). Metoprolol treatment reduced early and late trans-mitral flow velocities in iCEs, the E/A ratio, however, remained unchanged between baseline and metoprolol treatment (Fig. 8k–m). In line with lower HR, diastolic IVRT was prolonged after metoprolol treatment (Fig. 8n). Finally, velocity flow assessment of the LV outflow-tract demonstrated diminished color Doppler signals and corroborated decreased aortic peak velocities after metoprolol treatment (Fig. 8o, p). Viewed together, these findings mimic the proto-typic cardiac response to metoprolol to β-1 adrenoceptor signaling, indicating that *in-ovo* echocardiography is a suitable method to detect acute pharmacologically effects on cardiac physiology.

### Sub-chronic hypoxia exposure alters cardio-pulmonary flow profiles in iCEs

**Fig. 9:**
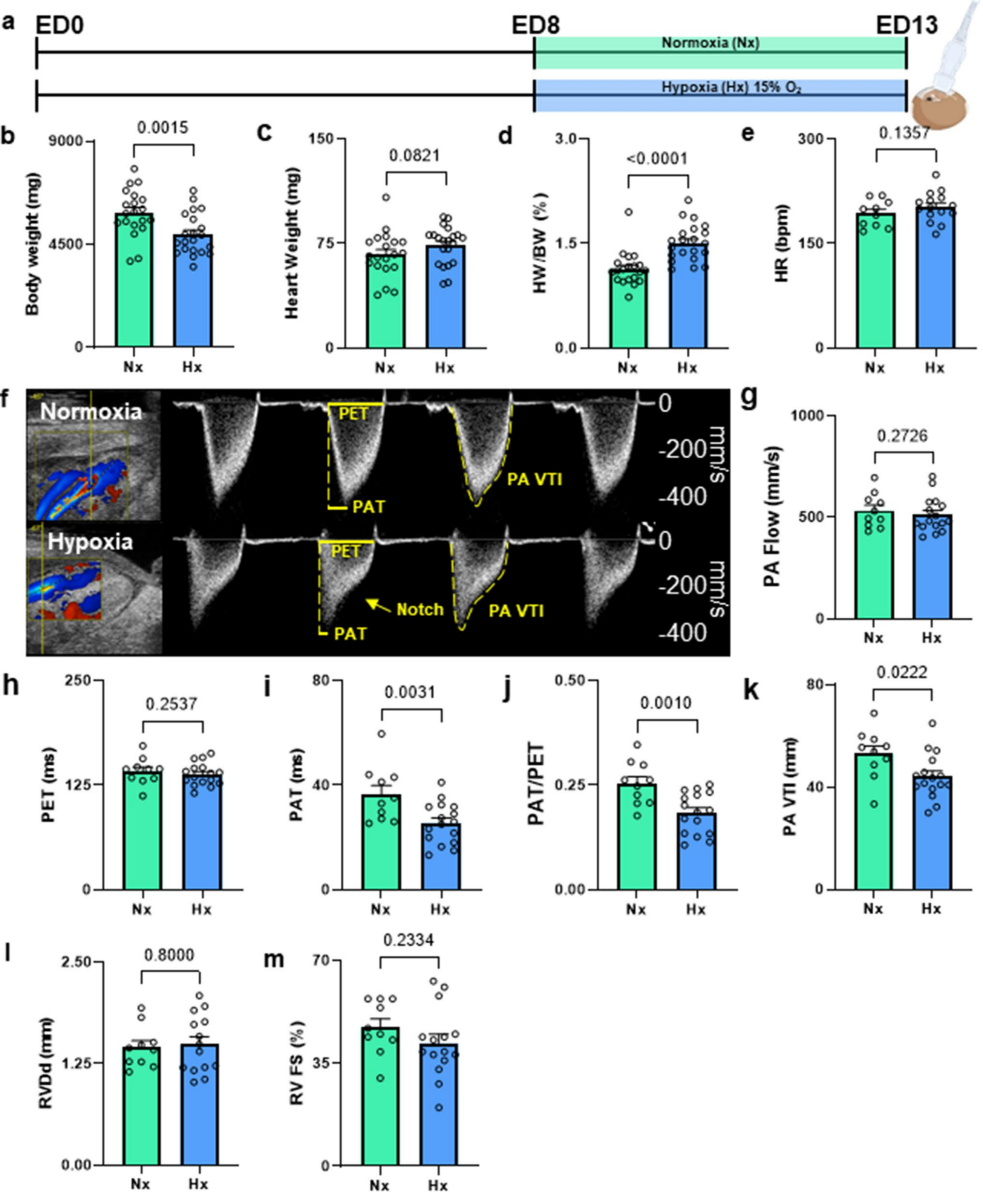
Gravimetric and echocardiographic evaluation of iCE after 5 days of hypoxic exposure. **a** Experimental outline. On ED 8, a subset of iCEs was placed into a hypoxic incubator with 15% O2. On ED 13, echocardiography was performed. **b** Total iCE body weight. **c** Total heart weight. **d** Heart weight (HW) to body weight (BW) ratio. **e** Heart rate (HR) in beats per minute (bpm). **f** Representative Color-Doppler and pulsed-wave Doppler images of the pulmonary artery flow in normoxic (Nx) and hypoxic (Hx) eggs. **g** Pulmonary artery (PA) flow. **h** Pulmonary ejection time (PET). **i** Pulmonary acceleration time (PAT). **j** PAT/PET ratio. **k** Pulmonary artery velocity time integral. **l** RV diastolic diameter (RVDd). **m** RV fractional shortening (RV FS). *N* = 20–21 per group for (**a**) and *N* = 10–16 for (**b–m**). Data are presented as mean ± SEM. Statistical analysis was performed by one-tailed unpaired student’s *t* test or Mann–Whitney test where applicable. *PET*  pulmonary ejection time, *PAT*  pulmonary acceleration time, *PA Peak Vel*   pulmonary peak velocity, *PA VTI*  PA velocity time integral, *RVDd *  RV diastolic diameter, *RV FS*   RV fractional shortening

To assess whether *in-ovo* echocardiography in iCEs would also be capable to detect structural and functional changes after exposure to sub-chronic interventions, we incubated iCEs in chronic hypoxia (15% O_2_, 1% CO_2_), a commonly used stimulus for induction of pulmonary hypertension in small animals, and probed for characteristic hypoxia-related changes in pulmonary blood flow by *in-ovo* echocardiography (Fig. 9a). After 5 days of hypoxia, body weight was lower in hypoxic vs. normoxic incubated iCEs (Fig. 9b), while heart weights tended to be higher in hypoxic iCEs compared to normoxic iCEs (Fig. 9c). Matching heart weight to body weight resulted in 30% increased heart-weight-to-body-weight ratios in hypoxic as compared to normoxic iCEs (Fig. 9d). For assessment of PA velocity profiles, we carefully controlled for HR in both groups to ensure comparability of normoxic and hypoxic iCEs (Fig. 9e). Flow profiles across the PA presented with distinct systolic notch morphology in hypoxic iCEs, a phenomenon that is clinically associated with increased pulmonary artery pressure[22] and deterioration of RV function in PH patients[1] (Fig. 9f). While peak PA flow velocities as well as pulmonary ejection time (PET) were—as expected—not significantly altered by hypoxia (Fig. 9g, h), the pulmonary hypertension marker pulmonary acceleration time (PAT) decreased by approx. 25% and, as a result, PAT/PET ratio decreased, comparable to measurements in hypoxic mice[40] (Fig. 9i, j). In addition, the PA VTI, a surrogate measurement for RV SV[4], was reduced, pointing towards reduced RV output (Fig. 9k), while RV diameter and FS were unchanged (Fig. 9l, m). Taken together, sub-chronic hypoxic exposure mimics features of PH pathophysiology in iCEs, well-known from hypoxia-based small animal models and PH patients [1, 22, 40], and can be reliably assessed using *in-ovo* echocardiography.

### *In-ovo* echocardiography demonstrates strong agreement rates between observers

**Fig. 10:**
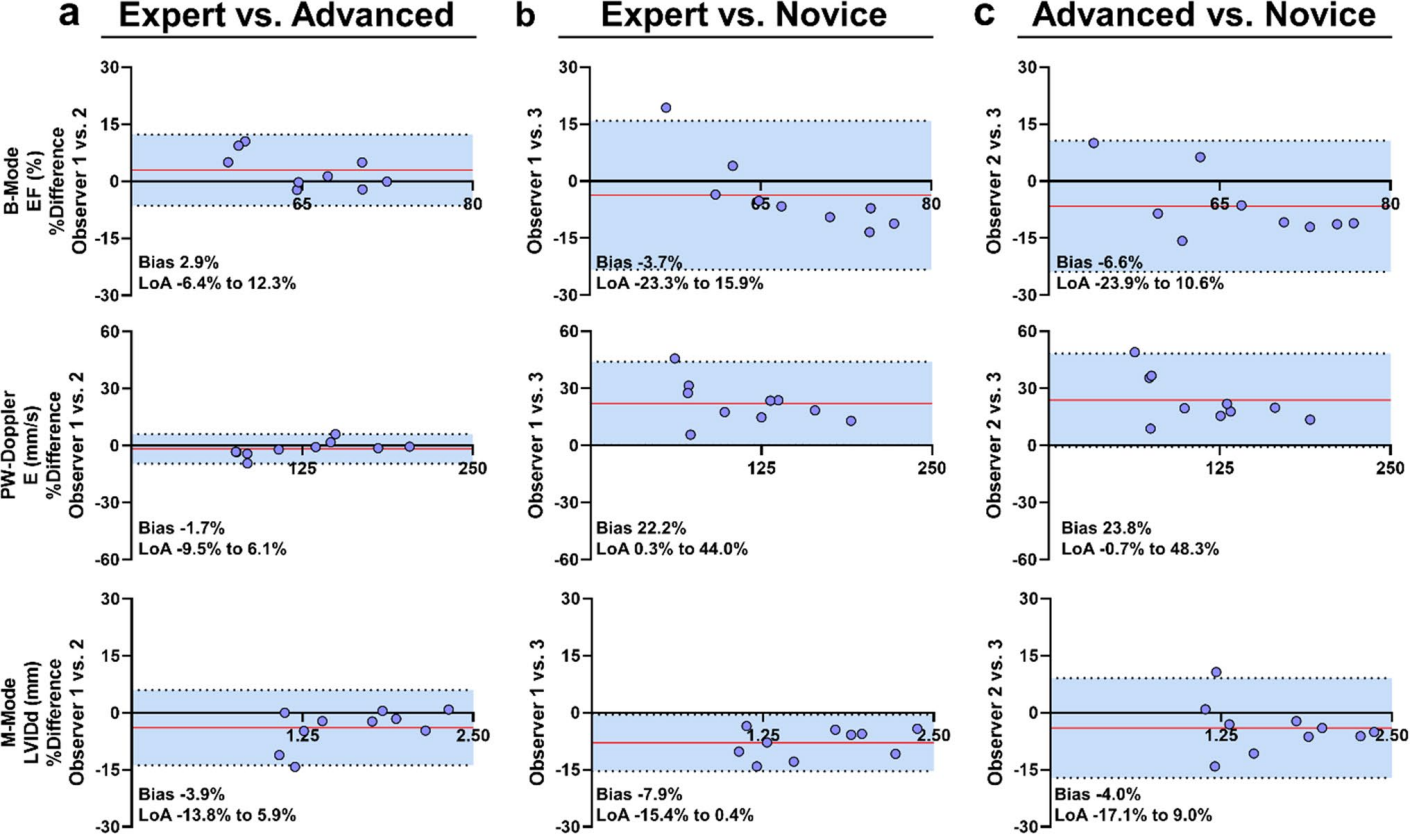
Echocardiography inter-observer analysis between three independent users. Inter-observer analysis was performed by Blant–Altmann analysis. Three independent echocardiography users of different training levels (observer 1: expert observer 2: advanced observer 3: novice) assessed images from iCEs at ED 9 and ED 13 for left ventricular (LV) ejection fraction (EF) from B-Mode images, early mitral inflow velocity (E) from pulsed wave (PW) Doppler recordings in the LV, and LV inner diameter in diastole (LVIDd) in M-Mode images of the LV (*N* = 10). **a** Comparison between expert and advanced user. **b** Comparison between expert and novice user. **c** Comparison between advanced and novice user. Statistical analysis was performed by Blant–Altmann plots with indication of observer bias and 95% limits of agreement (LoA)

Blant–Altmann analysis was performed to assess inter-observer agreement and variabilities between three independent users with different levels of expertise in echocardiography—observer 1: expert; observer 2: advanced; observer 3: novice (Fig. 10). From B-Mode, M-Mode and pulsed-wave Doppler measurements, we used one representative parameter each to assess observer agreements (B-Mode LV: EF, M-Mode: LV inner diastolic diameter (LVIDd), pulsed-wave Doppler: E wave), while a set of additional parameters can be found in the supplement (Supplement Table 2). Expert and advanced observers showed excellent agreement with minimal bias for EF (2.9%), E wave velocity ( – 1.7%) and LVIDd ( – 3.9%) (Fig. 10a). As expected, expert and novice generally showed lower agreement rates than expert and advanced observers, e.g., indicated by a tendency to overestimate E wave velocity by the novice observer (22.2%) (Fig. 10b). Results for advanced and novice user largely replicated these findings: LoA were larger than in the expert vs. advanced user comparison but bias was again low (EF:  – 6.6%; LVIDd:  – 4.0%) except for E wave velocities (23.8%) (Fig. 10c). As could be expected, reproducibility of *in-ovo* echocardiography hence relies on observer’s experience levels. Inter-observer agreement rates are comparable or even slightly higher in comparison with clinical [18] and small animal echocardiography [13] analyses, supporting the practicality of *in-ovo* echocardiography.

## Discussion

Here, we demonstrate that *in-ovo* echocardiography is a suitable tool to assess physiological and pathophysiological characteristics of cardiac structure and function in iCE. We provide reference values and a detailed protocol for this novel tool that guarantees high reproducibility and strong inter-observer agreement. We further demonstrate the sensitivity of *in-ovo* echocardiography by reproducing characteristic cardiac responses to sympatholytic and hypoxic interventions in iCEs.

The iCE has been used extensively in studies of tumor biology and angiogenesis [8, 29, 34] but has been rarely used in studies of cardiac physiology or disease. The few previous reports that introduced the idea to assess cardiac function by echocardiography in chicken eggs [2, 31] were severely limited by poor image quality and the lack of a methodological approach that covers the broad spectrum of measurements and ultrasonographic imaging modalities necessary to match rodent echocardiography. On this account, we are the first to conduct high-quality B- and M-Mode imaging of the whole heart from ED 8 to ED 13 in a modified apical imaging view comparable to the (pre-)clinically used apical four and five chamber imaging windows [25]. Moreover, Color-Doppler and pulsed-wave Doppler allowed us to assess velocity flow profiles in the left and right heart, as well as aorta and PA, completing the imaging repertoire of *in-ovo* echocardiography. Due to their overall size, cardiac dimensions and volumes are generally smaller in the iCE compared to small rodents, such as mice; however, relative functional metrices such as EF or FS proved to be comparable to small animals and humans, emphasizing the translational value of the *in-ovo* model [14, 15, 25]. Treatment with the sympatholytic β-1 adrenoceptor selective antagonist metoprolol phenocopied the negative chronotropic and inotropic effects known from mice and human studies [10, 26]. Sub-chronic hypoxic incubation yielded development of systolic notching of PA flow and a shortened PAT, both common hallmarks of PH detected in hypoxia-based mouse models and PH patients [1, 22, 40]. Consequently, PAT/PET ratio decreased as well, a validated non-invasive surrogate parameter of invasively measured RV pressures that accurately appraises PH severity in rodents [35]. Results on data reproducibility indicate overall low bias and decent observer agreement rates, in particular among experienced echocardiographers, which are partly superior to clinical and small animal studies [13, 18].

Novel *in-ovo* echocardiography complements the tool box of animal-experiment replacement methods, where ultrasonographic and high-speed video approaches, such as optical coherence tomography or semi-automated optical heartbeat analysis, have been used before to assess cardiac function and morphology of *Drosophila melanogaster* (*D. melanogaster*) and *Danio rerio*, the zebrafish [7, 11, 16, 30]. While these models are a viable and cost-effective alternative to actual animal experiments, iCEs present with superior anatomy and physiology [36]. The iCE has a four-chambered heart, equivalent to mammals, while zebrafish have a two-chambered heart and *D. melanogaste*r a tube-like heart with an open circulatory system. In addition, iCEs also have a respiratory system that is anatomically much closer to that of humans, allowing for the assessment of cardio-pulmonary interactions. As a consequence, the range of imageable parameters of the iCE’s heart is much greater compared to the zebrafish and *D. melanogaste*r due to its superior anatomical complexity and phylogenetic proximity to mammals. In fact, genetic similarities between chickens and humans are ~ 80%, compared to 75% between humans and zebrafish and 60% between human and *D. melanogaster* [19, 24], highlighting *in-ovo* echocardiography as the animal-free alternative method closest to mammalian physiology. The successful development of *in-ovo* echocardiography paves the way for its application in basic cardiovascular research and its contribution towards a 3R compliant research environment.

With the technical and biological feasibility of *in-ovo* echocardiography demonstrated, it is important to recognize its limitations as well as opportunities for the cardiovascular research community. A limitation of iCE usage is the absence of transgenic iCEs, hampering mechanistic approaches studying the role of specific genes, proteins or other molecules. While standard-of-use downstream assays as quantitative real time PCR and histology are feasible in iCEs, antibody-based assays such as flow cytometry and ELISAs are restricted by the limited availability of antibodies targeting chicken antigens. The latter may prevent the application of *in-ovo* echocardiography in the field of cardioimmunology. A main area of application may be high-throughput screening for potential cardiotoxic substances, due to fast data acquisition and low costs. Moreover, physiological changes in response to pathological stimuli as demonstrated by us for hypoxia may be studied using *in-ovo* echocardiography and will open the door for the testing of pharmacologic and therapeutic interventions. Overall, the potential of novel *in-ovo* echocardiography and its potential areas of application will be strictly defined by cardiovascular disease models developed by the basic research community in future.

Taken together, the present study provides evidence for the applicability and translational suitability of *in-ovo* echocardiography in cardiovascular research. Biventricular cardiac anatomy of iCEs can be reliably imaged using infrastructure that is commercially available and commonly used in small animal labs. Hence, *in-ovo* echocardiography is a suitable alternative tool to reduce and replace small animal numbers in pre-clinical cardiovascular research*.*

### Supplementary Information

Below is the link to the electronic supplementary material.

Supplementary file1 (PDF 656 KB) Supplementary Material 1: Standard Operating Procedure “in-ovo” Echocardiography

Supplementary file2 (XLSX 51 KB) Supplementary Material 2: Underlying data

Supplementary file3 (MP4 2457 KB)

Supplementary file4 (MP4 2616 KB)

Supplementary file5 (MP4 2557 KB)

Supplementary file6 (MP4 2582 KB)

Supplementary file7 (DOCX 1550 KB)

## Data Availability

Underlying data can be found in the supplement (Supplementary Material 2).
