# Supplementary material for: In-ovo echocardiography for application in cardiovascular research
Source: Basic Res Cardiol. 2023 May 16;118(1):19. doi: 10.1007/s00395-023-00989-0 (PMC10188421; doi:10.1007/s00395-023-00989-0)
Supplement: Supplementary file 1 — Supplementary file1 (PDF 656 KB) Supplementary Material 1: Standard Operating Procedure “in-ovo” Echocardiography [file 395_2023_989_MOESM1_ESM.pdf]

Standard Operating Procedure „*in-ovo*“ Echocardiography

Materials

Chicken Eggs

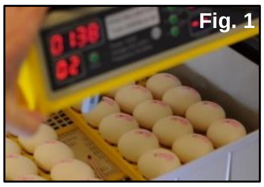

Fig. 1

- Fertilized, specifically pathogen-free eggs of white leghorn chicken (*Gallus gallus*)
- Incubation should be carried out in a humidified (<58%) egg incubator at 37.8°C (e.g. Janoel JN8-48; Fig. 1)

Equipment

Ultrasound unit

- Vevo3100 Workstation
- MX700 transducer
- Mounting/railing system (all Fujifilm Visuialsonics Inc., Canada)
- or equivalent ultrasound system

Other hardware

- Silicone mold with an oval recess for holding the egg in place during imaging (self-built)
- Infrared lamp (e.g. Carl Roth, article no. X435.1)
- Infrared thermometer (e.g. Carl Roth, article no. LY46.1)
- Gooseneck lamp (e.g. Carl Roth, article no. HNH3.1)

Egg preparation

- Surgical forceps (e.g. Fine Science Tools, catalogue no. 11051-10, 11006-12, 11000-12)
- 1x phospate buffered saline (PBS)

Software

- Vevo LAB, license required (Fujifilm Visuialsonics Inc., Canada)

Procedure

! CAUTION Experiments need to be performed accrding to the respective national animal welfare as well as institutional guidelines. Inform yourself on applying laws and regulations.

Preparations

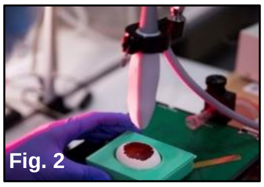

Fig. 2

- Turn on the Vevo3100 (or equivalent) ultrasound system and equip the MX700 transducer and choose the “Mouse Vascular” preset
- Attach the transducer head to the mount on the railing system (Fig. 2)
- Position the silicone mold underneath the transducer head
- Turn on the infrared lamp and aim it towards the silicone mold (approx. 50cm apart)
- Take an egg out of the incubator and place it into the mold
- Start to open the eggshell where the chorioallantoic membrane has detached
- Increase the opening as far as possible without harming the chorioallantoic membrane

+ **INSIGHT** You can use a gooseneck lamp to shine through the egg and better determine the localization of the chorioallantoic membrane and the embryo (Fig. 3)

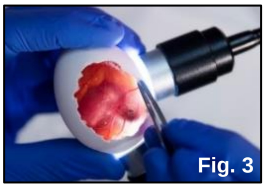

Fig. 3

- Using the infrared thermometer, assess the temperature on top of the chorioallantoic membrane, aiming at >34°C.
- Add 250µl of sterile and pre-warmed (37°C) PBS onto the chorioallantoic membrane to act as coupling medium and to prevent dehydration

Δ **CRITICAL** We highly recommend, that you keep time periods without external heating of the incubated chicken eggs during transportation or preparation as short as possible, as temperature variations will affect heart rates and consequently cardiac function

Imaging

- Inspect the position of the embryo and aim the transducer (usuallay at an angle of 10-15°) towards the suspected position of the sternum/heart
- Intitate “Scan” on the Vevo 3100 and adjust the depth of the ultrasound image to the maximum possible extend
- Use the micromanipulators on the railing system to perform an image-guided search for the heart
- Commonly, the heart is found be angled 45° to the head of the transducer, which is a good position to perform all measurements
- Further adjust the position of the transducer to visualize both ventricles and atria, as well as the aorta (called modified 5 chamber view, short ^{mod}5CV; Fig. 4a)
- Once a decent position is found, adjust brightness and contrast and record B-Mode cine loops, for the assessment of left ventricular volumes, dimensions and function.
- Afterwards, place an M-Mode across the ventricular plane and record cine loops for assessment of LV mass as well as cavity and wall dimensions (Fig. 4b)
- Then, place the Color Doppler across the heart and identify flow in the left and right ventricle, as well as the aorta (Fig. 4c+d)
- Using the pulsed wave Doppler, record flow profiles in the lower part of the left ventricle, right ventricle and aorta

∆ **CRITICAL** Always adjust the Doppler angle according to the flow plane! Usually flow angles for *in-ovo* echocardiography are 45°. You must not exceed an angle of 60°!

- For assessing RV dimensions and function, record M-Mode across the RV in the ^{mod}5CV (Fig. 5a)
- Afterwards, by rotating the ultrasound transducer and by using the micromaniuplators, adjust your image to visualize LV, RV and the PA (Fig. 5b)
- With help of the Color-Doppler, a strong flow signal should be found coming out of the RV within the PA, which can be recorded using the pulsed wave-Doppler for assessment of PA hemodynamics (Fig. 5c)

\+ **INSIGHT** Sticking to a standardized routine when acquiring imaging data (e.g. first LV imaging, then aorta, then RV, etc.) will reduce variation within your datasets.

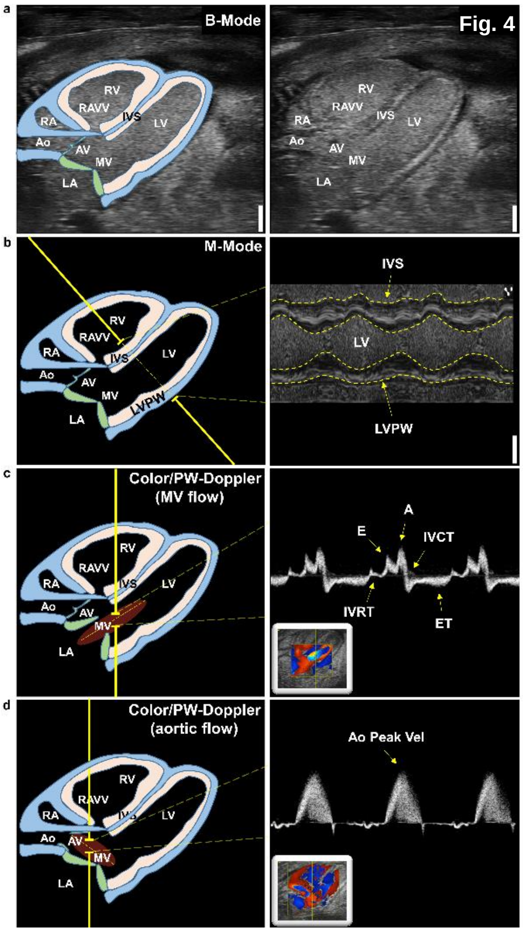

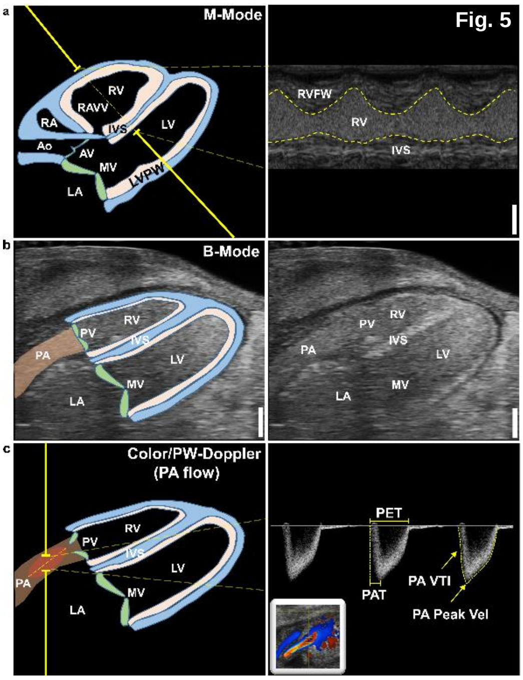

Analysis

- Perform offline analysis of your recordings according to common standards
- Fujifilm Visualsonics Inc. (www.visualsonics.com) offers a great variety of resources to learn about data analysis using the deciated Vevo LAB software (license required)
