# Supplementary material for: In-ovo echocardiography for application in cardiovascular research
Source: Basic Res Cardiol. 2023 May 16;118(1):19. doi: 10.1007/s00395-023-00989-0 (PMC10188421; doi:10.1007/s00395-023-00989-0)
Supplement: Supplementary file 7 — Supplementary file7 (DOCX 1550 KB) [file 395_2023_989_MOESM7_ESM.dcox]

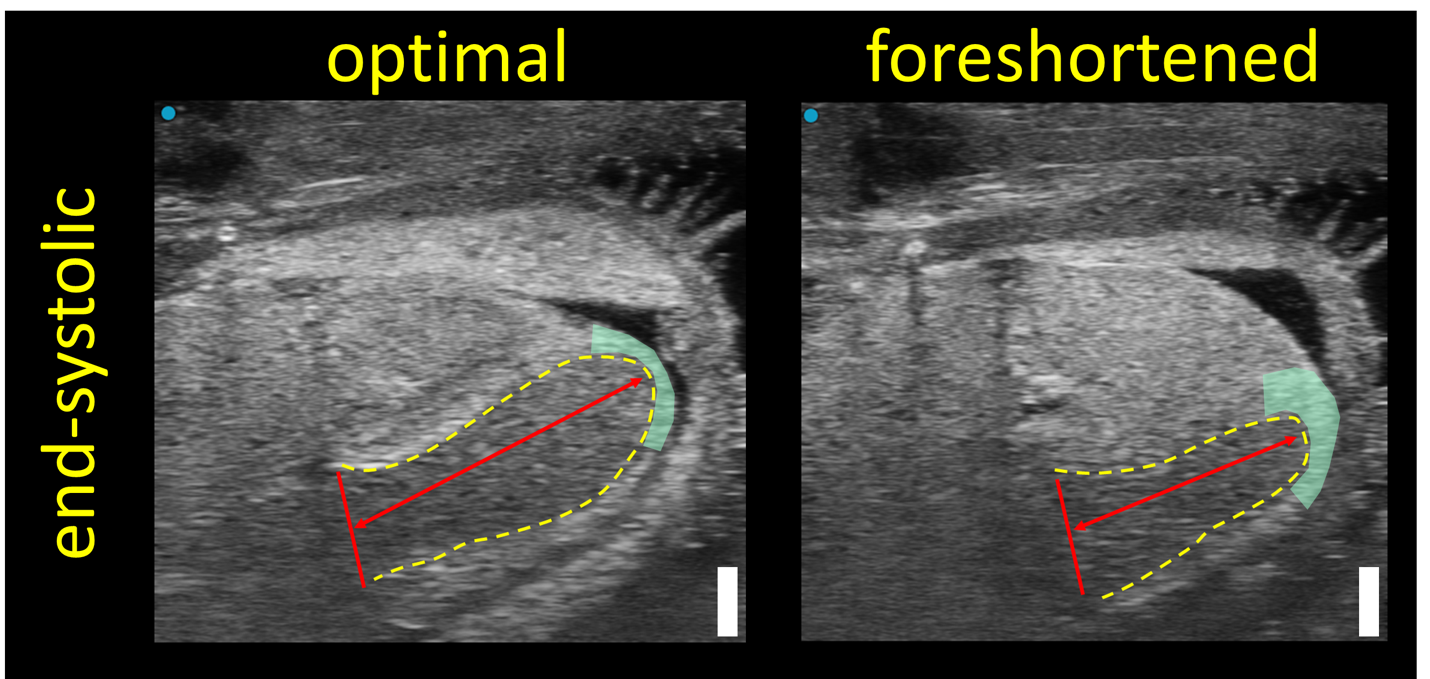


**Supplementary Figure 1: Foreshortening.** Compared with optimal images (left panel), foreshortened ventricles (right panel) have a reduced LV length (red arrow), smaller LV cavity (endocardium yellow dotted line) and show an apparent thickening of the apex (green area)[36]. Scale bar = 1 mm.


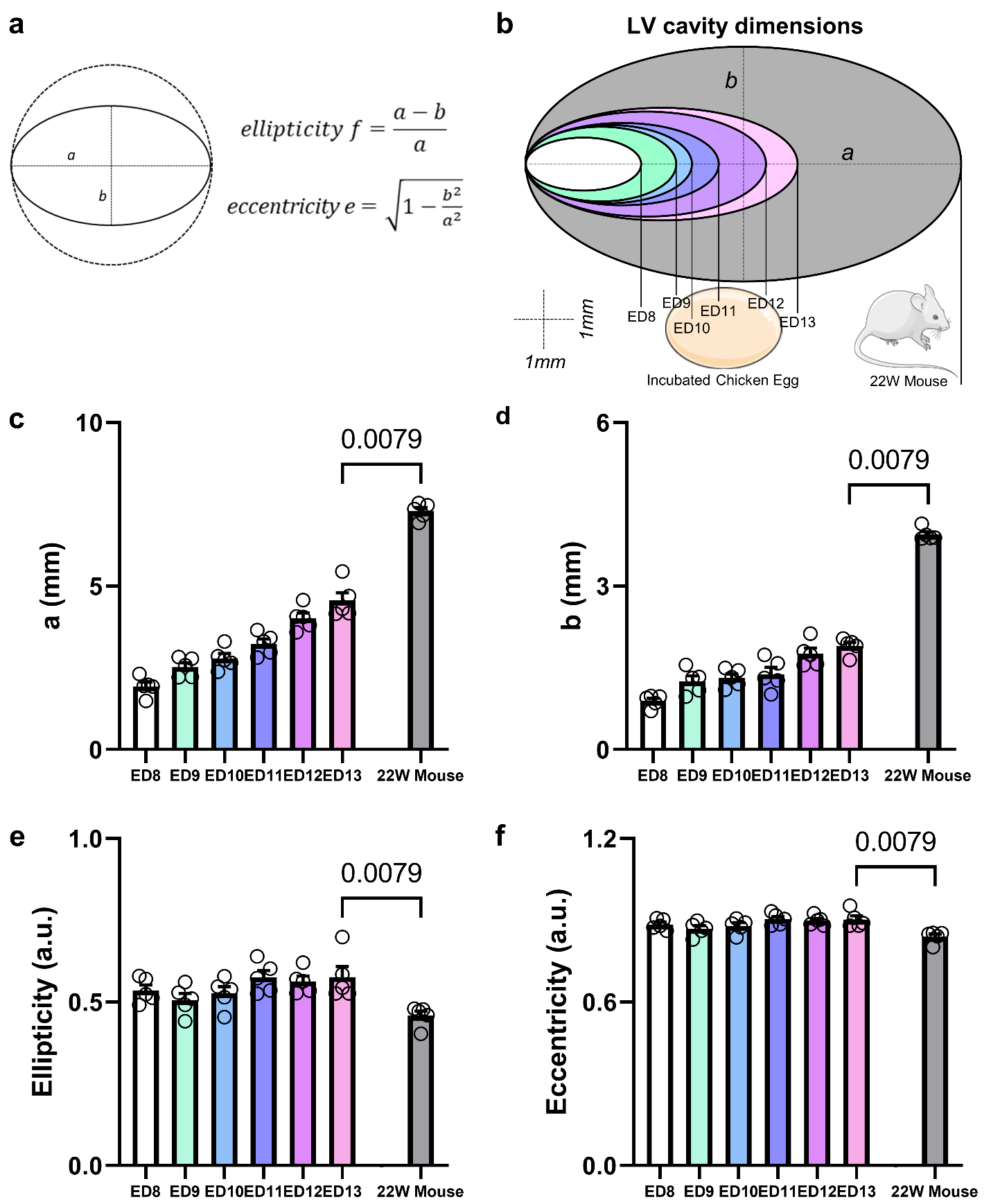


**Supplementary Figure 2: Assessment of left-ventricular geometry in iCEe vs adult mice. a)** Mathematic formulas to assess ellipticity and eccentricity of the LV. **b)** Average left-ventricular (LV) dimensions of iCEs on ED 8 to ED 13 and adult mice based on measurements of the end-diastolic LV cavity dimensions: distance from mitral valve (MV) to apex (*a*) and greatest vertical diameter (*b*). **c)** Measurements of the end-diastolic LV length from MV to apex (*a*). **d)** Measurements of the end-diastolic LV height from posterior wall to interventricular septum (*b*). **e)** Calculation of LV eccentricity. **f)** Calculation of LV ellipticity. Data are presented as mean ± SEM. Statistical analysis of iCE on ED 13 vs. adult mouse were performed by Mann-Whitney test.

**Supplementary Table 1: Comparison of common echocardiographic read-outs in healthy mice versus iCE.** Abbreviations: HR = heart rate (in beats per minute – bpm), ESV = end-systolic volume, EDV = end-diastolic volume, EF = left-ventricular ejection fraction, SV = stroke volume, CO = cardiac output, LVM = LV mass, LVIDd = inner LV diameter during diastole, LVAWd = thickness of the LV anterior wall during diastole, LVPWd = thickness of the LV posterior wall during diastole, W = Week, ED = embryonic day, m = male, f = female.


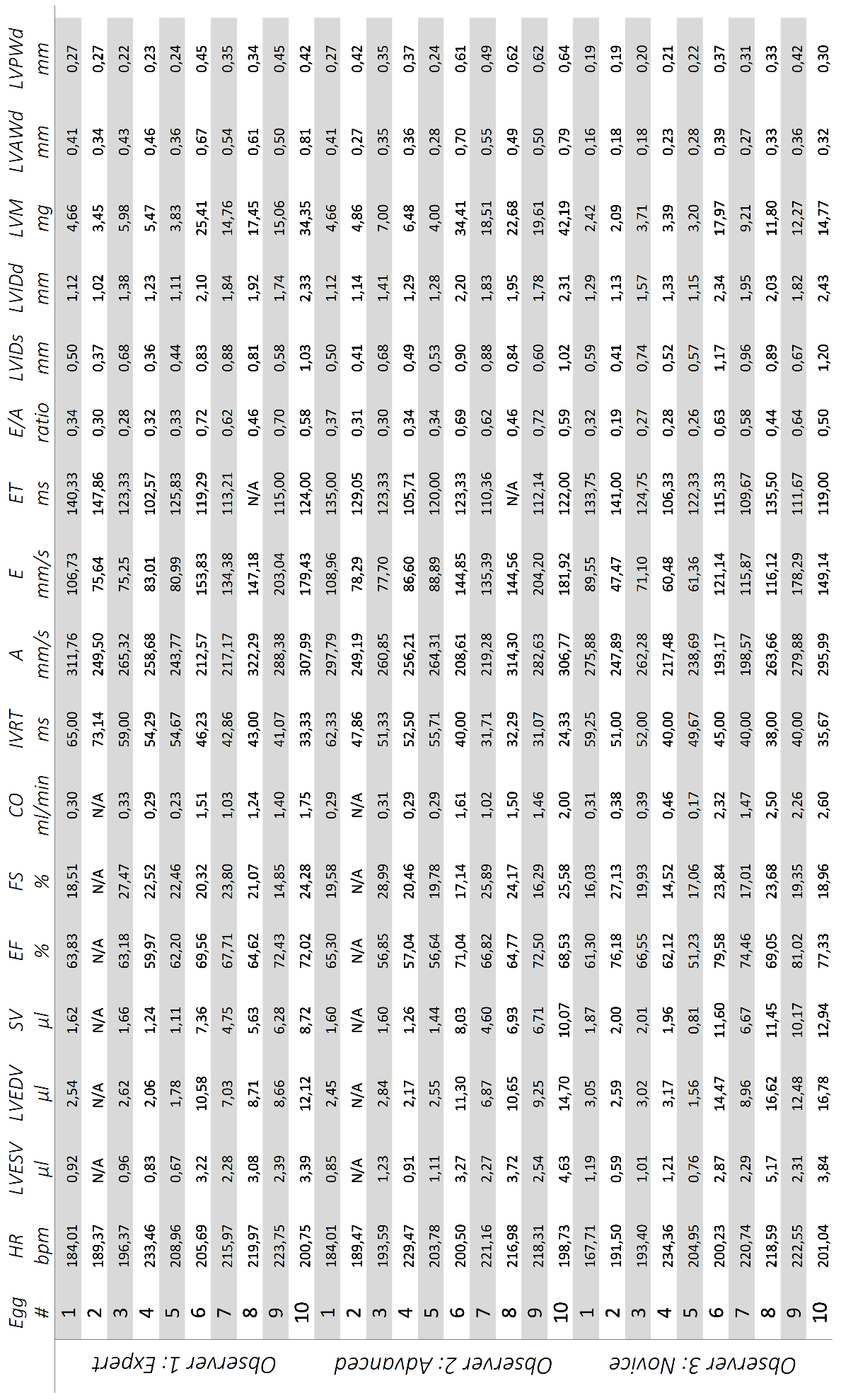


**Supplementary Table 2: Echocardiographic image analysis by three independent users of various expertise.** Abbreviations: HR = heart rate (in beats per minute – bpm), ESV = end-systolic volume, EDV = end-diastolic volume, EF = left-ventricular ejection fraction, SV = stroke volume, CO = cardiac output, LVM = LV mass, LVIDd = inner LV diameter during diastole, LVIDs = inner LV diameter in systole, LVAWd = thickness of the LV anterior wall in diastole, LVPWd = thickness of the LV posterior wall in diastole, LVESV = LV end-systolic volume, LVEDV = LV end-diastolic volume, IVRT = isovolumic relaxation time, E = early mitral inflow, A = late mitral inflow.
